# Dissociative symptoms in school-aged adopted children who experienced maternal separation and disruptive caregiving in infancy

**DOI:** 10.3389/fpsyt.2025.1453950

**Published:** 2025-04-17

**Authors:** Petra Winnette, Petr Bob, Ashley Datta

**Affiliations:** ^1^ First Faculty of Medicine, Charles University, Prague, Czechia; ^2^ Winnette Lab, Natama Institute, Prague, Czechia; ^3^ Center for Neuropsychiatric Research of Traumatic Stress, Department of Psychiatry and UHSL, First Faculty of Medicine, Charles University, Prague, Czechia; ^4^ Statistics Department, Columbia University, New York, NY, United States

**Keywords:** maternal separation, disruptive caregiving, stress, dissociation, behavioral problems

## Abstract

Amounting findings on maternal separation and early disturbed caregiving suggest that this type of early experience negatively influences socioemotional development and may be associated with behavioral and mental health problems in later life. Concerning previously published studies, we have assessed if maternal separation and disrupted caregiving before adoption in infancy could be related to heightened levels of dissociative symptoms and behavioral problems in middle childhood. We involved 30 children (sample S1) who had experienced maternal separation after birth and short-term institutional or foster care prior to adoption before 16.7 months of age. Based on the parents’ reports, they had not experienced any other significant adversities by the time of evaluation. These children were compared to a control group of children who have lived with their biological mothers in complete families (sample S2; *N* = 25). Although the findings are correlational and not causal, they indicate that specific adverse experiences, maternal separation after birth, and relatively short disruptive caregiving prior to successful adoption in infancy could be associated with significantly heightened levels of dissociative symptoms and behavioral problems in school-aged children. Our data also contribute to the literature on child socioemotional development and the etiology of dissociative disorders.

## 1 Introduction

### 1.1 Importance of caregiving and attachment relationships in infancy

In his attachment theory, John Bowlby postulated that an intimate bond between the mother and infant is necessary for healthy development and impacts the ability to form and maintain social relationships throughout the lifespan (1). Moreover, he concluded that a consistent and emotionally nurturing attachment relationship with the mother (or primary caregiver) is crucial for developing a cohesive sense of self and inner working model of relationships with others (2). Further research indeed confirmed that the primary caregiver, who is consistent and sensitive, presents an expected environment for the developing brain and the child’s healthy social and emotional functioning (3, 4).

### 1.2 Maternal separation and lack of stable attachment relationships

Extensive literature supports the attachment theory, implying that disturbed attachment relationships in infancy compromise social functioning and behavior later in life (5–7). Animal and human studies suggest that time-limited isolation of a pup or child from its mother is associated with long-lasting behavioral, psychological, and neurophysiological consequences and conclude that early stress related to impaired caregiving may negatively influence brain development and function and alter behavioral and neuroendocrinological stress responses in later life and adulthood (8–11). Early caregiving adversities may affect neuroendocrinological stress responses in childhood and adolescence via dysregulation of the hypothalamic–pituitary–adrenal axis, which may cause various cognitive and emotional deficits and negatively influence stress resilience, socioemotional functioning, behavior, and mental health (12–18). This way, theoretically, fragmented, inconsistent caregiving in infancy may present a specific early adverse childhood experience with long-term neurobiological consequences and deficits in multiple socioemotional domains (19).

Among these deficits are dissociative symptoms characterized by severe disruptions of mental integrity and personality development, which may lead to various psychopathological symptoms and behavioral problems, including psychotic and affective disturbances, as well as dissociation (20–22). Dissociation is associated with altering normal integrated functions of consciousness, memory, or identity as a response to an experienced traumatic event (21–23). Typical manifestations of dissociation involve memory loss, fragmentation of knowledge of the self and experience, splitting of emotional and/or cognitive aspects of experience, numbing of affect, psychological escape from unpleasant stimuli, trance-like states, and increased suggestibility (22, 23). In attachment literature, Hesse and Main (24) explored the relationship between disorganized attachment style and dissociative behaviors in infancy, such as infants being unresponsive, freezing, and avoiding eye contact. They suggest that disorganized attachment and these dissociative behaviors may result from the threatening behavior of the infant’s primary caregivers (24, 25). Meta-analyses concerning the effects of early attachment insecurity indicate that losses and disturbances in early attachment relationships have a lasting significant impact on psychosocial adaptation and externalizing behavioral problems (26). Behavioral problems refer to internalizing or externalizing behaviors, including withdrawal, somatic complaints, depression and anxiety, aggression, thought problems, attention deficits, inappropriate sexual conduct acting, and others, and often found in children with adverse experiences (27). The literature also discusses the links between dissociation and behavioral issues (48) and possible genetically based components of such correlation (82).

## 2 Current study objectives and hypothesis

The current study aims to contribute to the literature on early socioemotional development and dissociative disorders’ etiology by exploring the impact of a specific early caregiving situation on behavior and dissociation in a rare sample of children with unique care histories. The children in this study were abandoned by their mothers after birth and placed in a uniform and highly standardized childcare system in the Czech Republic (institutions or foster care), which provided good-quality physical and medical care. Before 16.7 months, they had all been successfully adopted and lived in stable families. Children in our sample, according to the mothers’ report, had not experienced physical abuse or neglect during infancy or later in childhood. They also had not experienced other significant adversities before evaluation (see the participant section for more details). However, they underwent the loss of their biological mother and a significant lack of consistent and sensitive primary caregivers in infancy, which severely impaired their chance to develop lasting attachment relationships during sensitive early social and emotional development. The current study examines if the loss of the mother after birth, followed by fragmented early caregiving without other significant adversities and traumas, may influence behavior and dissociative symptoms in school-aged children.

We hypothesized that the early traumatic event and stress experienced by infants due to maternal separation and caregiving disruptions prior to adoption would heighten the levels of dissociative symptoms and behavioral problems later in childhood compared to the control group of children who have lived with their biological mothers in complete families. Second, we were interested in whether the measures of dissociative symptoms and behavioral problems (CDC, BPM-M, BAC-C) are internally consistent and consistent with each other.

## 3 Participants and methods

### 3.1 Participants

In the present study, we included 30 children (sample S1) who experienced maternal separation as newborns, followed by pre-adoption care provided by a quality institution and foster care state system and adoption within 16.7 months. According to Mary Ainsworth, there are four general phases in developing early attachment relationships: 0–14 months, and after approximately 15 months, children typically start developing a wider range of social ties (83, 84). We intentionally aimed to involve children who were in substitute care for a limited period before adoption, starting at birth and ending at a maximum of 18 months. The rationale behind this decision was that at approximately 18 months, the infants’ socioemotional development continues toward more significant independence from the primary caregivers and involves managing a broader spectrum of relationships. This process means separating close-attachment relationships with primary caregivers and a wider circle of social ties (84). We wanted to focus on the early periods when primary caregivers were still essential social partners.

The participants’ ages ranged from 6 to 8.7 years (mean = 7.4, SD = 0.81), with 14 female and 16 male children. The age of adoption ranged from 12 to 73 weeks. Since the adoption, all children lived with their adoptive parents, with no further involuntary separation from them. The control group involved 25 children (sample S2) who had lived with their biological parents since birth and had not experienced any involuntary separation from them. Their ages ranged from 6 to 8.8 years (mean age = 7.3, SD = 0.83), with 13 female and 12 male children.

The highly standardized system in the Czech Republic consistently provides high-quality, comprehensive pre-adoption care, addressing infants’ physical needs, nutrition, medical attention, and opportunities for play and interaction with other children. Children in care receive superior medical attention (29). The care is regulated by law and is subject to rigorous quality inspections. In these institutions, children are cared for by trained and supervised caretakers. However, these caretakers rotate shifts and care for eight infants simultaneously (28). Children in foster care are placed with quality state-run foster families that receive financial support and supervision (28). However, such placements are temporary solutions, and children undergo another significant change in caregivers upon adoption.

### 3.2 Inclusion/exclusion criteria

Our goal was to examine relatively homogeneous samples. Therefore, our design applied strict inclusion/exclusion criteria to minimize the impact of confounding variables [for example, children experiencing other traumas and showing mental health issues (clinical population) and cognitive developmental problems]. Mothers were first interviewed by phone and then provided information about their children and themselves in a structured care history questionnaire, which we adapted from structured anamnestic questionnaires used in clinical settings (30). The structured care history questionnaire addressed topics such as the child’s traumatic experiences and abuse, hospitalizations lasting more than 2 weeks without a caregiver, involuntary separation, traumatizing parental separation or divorce, and custody issues. We inquired about officially diagnosed mental health conditions, including ADHD, serious illnesses, and head injuries. Additionally, we asked about their involvement in the standard educational system. We excluded children who had such issues or required special educational support.

We only included children who were abandoned at birth and immediately placed either in good-quality institutions or in good-quality foster care. Their adoption age ranged from 3 to 16.7 months, and they had not been involuntarily separated from their adoptive parents since adoption (sample 1). We only included in the comparison group children who had lived with their biological parents since birth and had not been involuntarily separated from them (sample 2). The participants lived in different regions of the Czech Republic. According to the mothers’ responses in the questionnaire, the children in the study had not experienced significant adversities. They were neither formally diagnosed with significant mental health issues. All children attended a regular educational system and did not need special education programs at the time of the study.

#### 3.2.1 Primary caregivers

All adoptive mothers (and parents) underwent screening of their socioeconomic status, evaluation by a psychologist, and training by state-licensed professionals before adoption, as required in the compulsory adoption process in the Czech Republic. In the care history questionnaire, we asked about the mother’s education, traumatic events, marital status, serious illness, or other severe conditions within the last 3 years before the study. All mothers had high school or university levels of education. None of the mothers had been formally diagnosed with mental illness. We note that information about mothers was obtained via their reports in the care history questionnaire, and the answers may be biased. In addition, to minimize the impact on the possible mother’s current mental health issues, we administered General Anxiety Disorder-7 (GAD-7) and Patient Health Questionnaire-9 (PHQ-9). None of the mothers (and one father) showed elevated levels of anxiety or mental health issues at the time of the examination measured by the GAD-7 questionnaire (31, 32) and PHQ-9 (33, 34).

Both samples included consecutive participants obtained via advertising at websites, parent organizations, schools, and social services institutions across all regions in the Czech Republic. However, our sampling was not probabilistic because we aimed to include extremely hard-to-reach groups. We also note that we could not evaluate subtle parenting and detailed care history differences in the adopted or control samples.

All mothers or adoptive mothers gave written informed consent, and the study design followed all the criteria of the Declaration of Helsinki. The research was approved by the Ethics Committee of the General Charles University Hospital in Prague, number 979/20 OS-IV.

### 3.3 Psychometric measures

To examine the specific problem behaviors related to the traumatic attachment experience, we used the Brief Assessments Checklist for Children 4–11 (BAC-C). The BAC-C is a psychometric tool specifically constructed to evaluate attachment and trauma difficulties among adopted and fostered children with adverse caregiving histories (35). BAC-C has been widely used in studies focusing on children with institutional or foster-rearing histories. For example, a recent European study in Holland reported that the BAC-C is a valid and valuable screening measure for use with children in foster care. The internal consistency of the BAC-C was (Cronbach’s alpha = 0.89) (23).

BAC-C is a 20-item caregiver-report rating scale for clinically significant traumas and attachment-related mental health issues (25, 31, 35). The tool’s psychometrics have been confirmed (35, 36). The BAC-C questionnaire was translated into Czech using the standard procedure for validated translation of psychological questionnaires and tests (37).

The internal consistency of the BAC-C in this sample is Cronbach’s alpha = 0.894. The Czech version of the BAC-C internal consistency used in a follow-up study (72 participants) was also good (Cronbach’s alpha = 0.89 [0.84–0.92]) (19).

To examine general behavioral problems, we used the Brief Problem Monitor–Parent Form (BPM-P) for ages 6–18. BPM-P consists of 19 items scored by parents focused on problem behaviors of children (33, 36, 38). The BPM-P questionnaire is a shorter version of the Child Behavioral Check List 6–18 and has high internal consistency (Cronbach’s alpha = 0.91). For example, a recent study in Norway showed that BPM-P has good internal reliability and convergent validity (39). The BPM-P questionnaire was translated from English to Czech using the standard procedure required by ASEBA (37, 40–42). ASEBA approved the final translation as culturally and linguistically valid and registered it as the official Czech translation of BPM-P. Psychometric properties were established in previous studies (36, 43). The internal consistency of the BPM-P in this sample is very good (Cronbach’s alpha = 0.917). The Czech version of BPM-P used in a follow-up study (number of participants was 72) showed excellent internal consistency (Cronbach’s alpha = 0.93 [0.91–0.95]) (19).

We used the Child Dissociative Checklist (CDC) to assess dissociative symptoms in children (40, 41, 43). The CDC consists of 20 items scored by parents. Previous studies show that maltreatment during childhood, especially neglect and abuse, results in the development of dissociative behaviors (44–47). We used the official Czech translation of the CDC questionnaire (48). Psychometric properties (i.e., test–retest reliability and discriminant validity) have been established in previous studies (43, 45). The Czech version of the CDC tool has high internal consistency (Cronbach’s alpha = 0.86) (48). The internal consistency of the CDC in this sample is good (Cronbach’s alpha = 0.833).

Please note that partial data from this study were used after more subjects (*N* = 72) were included in the follow-up study. The follow-up study examined three separate groups (institutionalized children, fostered children, and comparison group) and studied behavior, dissociation, and empathy behaviors (19). We also gathered data on socioemotional development and attachment behaviors with the mothers through in-lab experiments. This information will be used in another article currently in preparation.

### 3.4 Statistical analysis

The statistical evaluation of psychometric measures’ results included means and standard deviations. Because stress and dissociative symptoms frequently do not have a normal distribution, we have used non-parametric Spearman correlation coefficients and non-parametric Mann–Whitney tests. In addition, we wanted to check how the length of pre-adoption care may affect stress and dissociative symptoms among the participants. To facilitate such an analysis, we have also divided our sample S1 into two subsamples (S1.1 and S1.2) based on the length of pre-adoption care (S1.1—less than 32 weeks with a sample size of 12 vs. S1.2—more than 32 weeks with a sample size of 18) and performed a similar analysis. To check the reliability of our measure, we have used multiple linear regression to assess childhood dissociation measured by the CDC in its relationship to behavioral problems (BAC-C and BPM-P). All statistical evaluation methods were performed using the software package Statistica version 10 and RStudio Build 351.

## 4 Results

Based on the mothers’ reports in the structured care history questionnaires, the adopted children included in the study had not experienced significant trauma or adverse childhood experiences other than maternal separation and disruptive caregiving in infancy prior to adoption (sample 1). According to the mothers’ reports, the never-adopted children had not experienced significant trauma or adverse childhood experiences (sample 2). The samples also do not differ in other measured parameters, such as involuntary separation or mothers’ mental health and education (see more details in the participants section). The maternal separation and disturbances in caregiving in infancy may present a meaningful difference between the samples.

**Figure 1:**
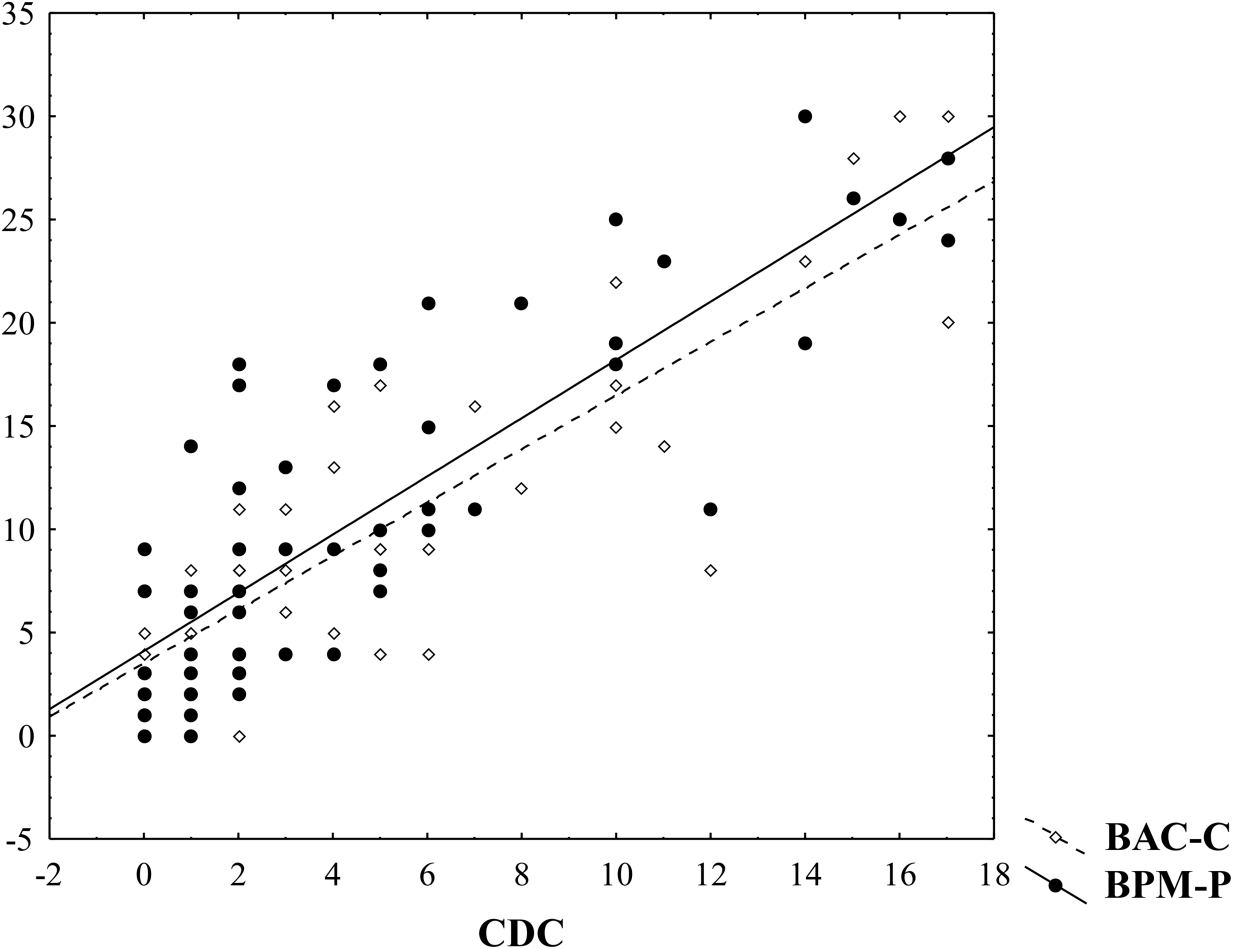
Relationships of child dissociation measured by CDC with psychometric measures of behavioral problems (BAC-C; BPM-P). The Spearman correlation between CDC and BAC-C and between CDC and BPM-P for the whole sample (S1+S2) is *R* = 0.81, *p <*0.01 and *R* = 0.82, *p <*0.01, respectively.

The first hypothesis we want to test is whether maternal separation and disrupted caregiving at infancy lead to a higher level of behavioral problems and dissociative symptoms compared to never-adopted children in the control group. Our results show that the participants who experienced maternal separation and disruptive caregiving in infancy manifest significantly higher levels of dissociative symptoms and behavioral problems (**Tables 1**, **2**, **Figure 1**). **Table 1** shows that based on the effect size (Cohen’s *d*) found in the data, we needed approximately 14 participants in each sample (S1 and S2) to detect the difference in the data with 80% power. Our sample size (30 + 25) is enough for this study.

**Table 1:** Statistical analysis describing descriptive results of psychometric measures and statistical differences using the Mann–Whitney test between subgroups 1 and 2 (S1 and S2) with *p <*0.01 for all statistical comparisons.

	Mean S1	SD S1	Mean S2	SD S2	Mann–Whitney *U*	*Z*	*p*	*t*-test	*p*	Cohen’s *d*	Sample size
BAC-C	13.23	7.92	5.08	3.05	115.00	4.39	0.000011	4.85	0.000011	1.31	11
BPM-P	14.37	8.89	6.16	4.68	171.00	3.45	0.000564	4.16	0.000119	1.13	14
CDC	7.17	5.32	1.60	1.73	110.50	4.47	0.000008	5.01	0.000006	1.36	10

**Table 2:** Statistical analysis describing Spearman correlation coefficients of psychometric measures for subgroups S1 and S2 with *p <*0.01 for all statistical comparisons.

	BAC-C	S1 + S2	CDC	BAC-C	S1	CDC	BAC-C	S2	CDC
		BPM-P			BPM-P			BPM-P	
BAC-C	1.00	0.82	0.81	1.00	0.89	0.81	1.00	0.64	0.57
BPM-P	0.82	1.00	0.82	0.89	1.00	0.87	0.64	1.00	0.58
CDC	0.81	0.82	1.00	0.81	0.87	1.00	0.57	0.58	1.00

**Figure 2:**
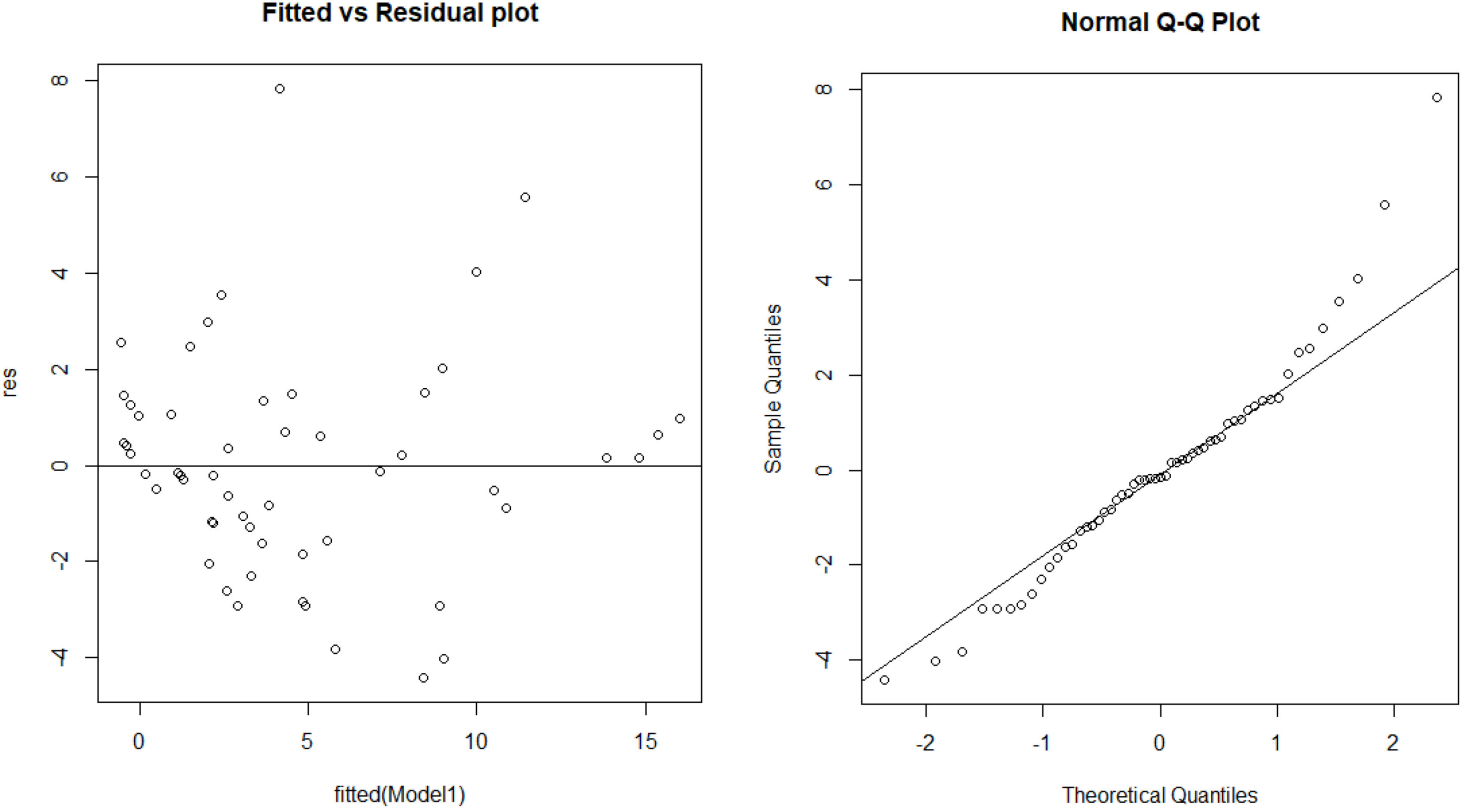
The fitted vs. the residual plot and the QQ plot of regressing child dissociation measured by CDC with psychometric measures of behavioral problems (BAC-C; BPM-P).

Second, we wanted to confirm whether the measures of dissociative symptoms and behavioral problems (CDC, BPM-M, BAC-C) are internally consistent and consistent with each other. To check our measures’ internal consistency, we calculated Cronbach’s alpha (CDC: 0.833, BAC-C: 0.894, BPM-M: 0.917) for each of the three measures, showing a high internal consistency level. Our results also suggest that dissociative symptoms measured by the CDC manifest significant correlations with other psychometric and behavioral measures. For example, in the entire sample (S1+S2), the Spearman coefficient in between any two of the measures turns out to be in the 0.81–0.82 range (see **Table 2**). In **Table 2**, we also see that the Spearman coefficients in between the measures are higher in sample S1 than in sample S2. Considering that sample S1 tends to have higher values than sample S2, this may be due to those measures closely aligning with one another when they detect some psychometric and behavioral abnormalities. Cronbach’s alpha in between the three measures calculated from our sample (S1+S2) is 0.921, which shows that the CDC measure has high consistency with the other two measures (BPM-M and BAC-C). In addition, the results of multiple linear regression suggest that childhood dissociation measured by the CDC is closely related to behavioral problems measured by BAC-C and BPM-P (*R* = 0.89; adjusted *R* = 0.88; *F* = 96.4; BAC-C beta = 0.38; BPM-P beta = 0.22). The VIF of 3.53 (>3) between BAC-C and BPM-P indicates a moderate correlation. In **Figure 2**, the fitted vs. residual plot along with the normal QQ plot of the residual is provided. The Shapiro–Wilk normality test for residuals gives a *p*-value of 0.04206, indicating slightly non-normal residuals. Due to this, our analysis primarily consists of non-parametric tests, which do not assume the normality assumption.

Given that the literature suggests that time spent in institutional or foster care may play a role in later socioemotional development, we conducted an additional exploratory analysis to test whether the length of maternal separation at infancy affects the extent of behavioral problems. Our results in **Table 3** fail to find any significant difference in dissociative symptoms and behavioral problems between the two subgroups (S1.1 and S1.2) based on the length of pre-adoption care. A larger sample size might be necessary to detect if such a difference exists (**Table 3**). We calculated Cohen’s *d* based on the data, and it indicates a very small effect size and thus requires many samples (>300 per group) to be able to detect such a small change between samples S1.1 and S1.2 (**Table 3**).

**Table 3:** Statistical analysis describing descriptive results of psychometric measures and statistical differences using the Mann–Whitney test between subgroups 1.1 and 1.2 (S1.1 and S1.2).

	Mean S1.1	SD S1.1	Mean S1.2	SD S1.2	Mann–Whitney *U*	*p*-value	Cohen’s *d*	Sample size (per group) required (80% power)
BAC-C	12.25	9.37	13.89	7.00	86.5	0.3721	0.204	379
BPM-P	13.25	8.75	15.11	9.16	97.0	0.6563	0.207	368
CDC	7.42	5.53	7.00	5.32	116.0	0.7497	0.077	2,649

The results indicate no significant difference between subgroups 1.1 and 1.2.

## 5 Discussion

Most studies conclude that early disturbed caregiving hurts socioemotional development and the ability to form and maintain attachment relationships. However, what types of early adverse childhood experiences specifically contribute to the dissociative symptoms remain unclear (47). The current study provides unique and more specific data in this context. We present cross-sectional findings focused on the associations between the separation of children from their mothers immediately after birth, followed by limited time in pre-adoption care (3–16.7 months) before they were placed with stable adoptive parents and behavioral and dissociative problems later in childhood. Although the children examined in this study had presumably all appropriate physical and medical care in pre-adoption care in infancy and, to our knowledge, they had no other significant adversities after adoption, their dissociative symptoms and behavioral problems appear to be significantly higher than those in the control group. The data show significant effects in all measured variables, indicating that the newborn’s separation from its mother and disturbed caregiving in infancy may later negatively influence dissociative symptoms and behavioral problems.

### 5.1 Impact of adversities in childhood on behavior and dissociative symptoms

Earlier studies report that maltreatment during childhood, especially neglect and abuse, strongly contributes to the development of dissociative behaviors (44, 49–51). Specifically, research on child abuse and neglect implies that maltreatment in caregiving relationships predisposes children to complex mental health issues (16, 52, 53). Children who experience complex developmental trauma often show heightened levels of dissociative symptoms (35, 54). Social rejection can also hinder the development of healthy self-knowledge, resulting in dissociative symptoms and related disturbed behaviors concerning other people (55). The literature indicates a significant association between caregiving and family environment and later dissociation, especially when the children experienced a lack of warmth and safe emotional connection with primary caregivers compared to the non-patient population (57). Moreover, disturbed relationships with caregivers on their own can impact dissociative symptoms independently of other types of maltreatment (47, 58).

### 5.2 Adopted children and early caregiving

Studying adopted children presents an opportunity to explore how changes in caregiving history influence social and emotional development and mental health issues later in childhood. At the same time, adopted children represent a rather heterogeneous population; some exhibit resilience, while others experience more severe challenges (7). Previous research indicates that although successfully adopted children generally function well in certain areas, such as attachment behaviors, physical growth, and cognitive functioning (56, 58–60), they face a greater risk of behavioral problems than children who have always lived with their biological families (61–63). The literature also suggests that children adopted early in infancy often catch up in some areas of development better than children adopted later (81). However, the current study findings surprisingly did not support these results. Children in our sample who spent 32 to 64 weeks in care did not show significantly higher scores in problem behavior or dissociation compared to those who spent 0 to 32 weeks in care. One explanation could be that many studies, including previously institutionalized and fostered children, had fairly limited or no information regarding the children’s history with their biological parents before placement, as well as significant differences in the quality of care in institutions or foster settings (60, 64). In such instances, the differences may reflect the wide range of early experiences and adversities children in our samples did not encounter to the same degree. However, our samples are too small to draw definitive conclusions, and the results should be regarded as exploratory.

Although most adoptees differ considerably in many domains of development (4, 64), they typically undergo periods with considerably dysfunctional parents who provide disorienting social interactions and impaired care. As a result of that, the child is removed from the biological family and experiences a loss of attachment relationships and deep relational trauma. That is often followed by temporary placement in care and consequent adoption into a new family. Although adoption presumably presents a positive outcome, altogether, such a trajectory is a severely disconcerting experience for a child. John Bowlby postulated that such early experiences may induce the disintegration of the “self” and negatively influence the development of the internal working model (65). Main and Solomon (66) suggested that such parental behavior in infancy strongly contributes to the disorganized quality of the child’s early attachment relationship with them. Lioti and Tucker (67) emphasized that early disorganized attachment relationships and later traumas equally present a high risk for the later development of dissociation. Meta-analytic studies agree that the disorganized quality of attachment relationships in infancy is associated with parental psychopathology, maltreatment, and pathological interactions with the child and leads to significant behavioral problems (68).

Dissociation in non-clinical populations has been researched in adolescents and adults (69). Fewer studies in children report that maltreated children show higher scores of dissociation behaviors than healthily developing children. For example, Macfie et al. (49) studied 4-year-old maltreated and non-clinical children. They found that although non-clinical groups had dissociative symptoms, the maltreated group showed significantly higher scores. In their longitudinal study, which included non-clinical participants, Ogawa et al. (70) confirmed that the poor quality of early attachment relationships with caregivers during the first 18 months, especially the primary caregiver’s unavailability, strongly contributes to dissociation later in life. The literature overall agrees that although the non-clinic population may show dissociative symptoms, especially in high-stress situations, clinical populations and those with a history of childhood traumas show significantly higher dissociative scores.

However, there are still gaps in the overall understanding of the development and etiology of dissociative symptoms (38, 71, 72). One of the significant limitations of the studies focused on the relationship between dissociative symptoms and trauma is that it is challenging to untangle the impact of developmental issues, such as early attachment relationships, developmental trauma, and later incidents of abuse or other traumatic experiences.

In this context, the present study’s findings are congruent with results describing maternal separation in animals, indicating that the early stress experiences caused by maternal separation influence stress responses and behavior in rodents and subhuman primates and have long-term effects on behavioral and endocrine dysfunctions (11, 73). Regarding the current research in humans, the results of the present study are in agreement with the findings indicating that dissociation, described as a disruption of integrated mental functions, usually manifests due to child abuse or neglect (74) and insufficient parental caregiving or emotional deprivation (67, 75–80). Interestingly, the time the children spent in the institutional care of foster care (ranging from 0 to 32 weeks or 32 to 64 weeks) did not make a significant difference (81). The present study’s findings in children confirm that the early presence of a stable primary caregiver, consistent care, and a chance to develop a stable attachment relationship is critical to healthy development. Notably, the strong correlation between behavior and dissociative symptoms suggests that the issues in socioemotional development within our samples may be closely intertwined. This finding highlights the need for differential diagnostic assessment in clinical settings (48) and future research to explore those relationships. We also recognize that dissociative symptoms may be connected to broader psychopathological concerns that exhibit stability throughout early childhood, later childhood, and adolescence. This relationship may be associated with genetic factors (82). However, we could not examine this possibility in the current study, as we could not access information about the participants’ biological parents and their extended biological family. On the other hand, our findings indicate that maternal separation and inconsistent primary caregiving in infancy may present specific adverse childhood experiences in early emotional and social development with long-term consequences.

### 5.3 Limitations

The present study has limitations, and future studies should address the following factors.

1. Given our efforts to assess considerable homogeneous groups of children with unique caregiving histories, our sample was relatively small.
2. We could not learn about prenatal development and the children’s biological parents. Further studies utilizing genetically informed designs should help disentangle the impact of these variables.
3. We have not assessed certain parental aspects that may have influenced the overall development of the children in our samples (including details of their socioeconomic status, genetic influences, and more about post-adoption parent–child relationships). These factors should be included in future studies.
4. We acknowledge that all information was obtained through the parents’ reports in the questionnaires, which may introduce bias. Future research should include methods that complement parental information with direct medical, neurobiological, and behavioral data.
5. Given that we realized comprehensive language-validated translations of the BAC-C and BPM-P, which were officially accepted as valid by ASEBA and the author of BAC-C, and those had been commonly used in Europe in similar cultural contexts, we did not run the empirical validation for this study. The validation of these valuable measures for the Czech context should be addressed in further studies.
6. The participants were recruited through information leaflets and website information disseminated by schools, preschool programs, social services, and parent organizations. However, our sampling was not probabilistic as we aimed to include relatively homogeneous, extremely hard-to-reach samples of children with unique care histories.
7. We also note that we could not evaluate subtle differences in parenting and care history either in the adopted or control samples, which, however, naturally influenced the children’s overall social and emotional functioning in both samples.

## 6 Conclusions and directions for future research

In summary, the findings of this study suggest that maternal separation after birth and disruptive caregiving in infancy, experienced by children in sample 1, could later be associated with increased levels of dissociative symptoms and behavioral issues compared to the control group. These children presumably received adequate pre-adoption care, including suitable physical and medical attention, and, to our knowledge, did not encounter any other significant traumas or adversities after adoption. The data suggest that such early experiences could contribute to impaired social and emotional development, thereby raising the risk of mental health issues later in childhood. Our findings also provide new insights into the etiology of dissociative symptoms.

The results may inform policymakers responsible for designing infant childcare facilities and services, emphasizing the role of individual, consistent, and sensitive caregiving. Our data can also enrich parents’ education, with emphasis on future adoptive parents, about the importance of involved and consistent parenting in early development for children’s further mental health (65). Examining specific adversities associated with early inconsistent caregiving (19) would also benefit clinical work with patients, children, and adults suffering dissociation and problem behaviors in relationships and support developmentally oriented therapeutic modalities that focus on the impact of early socioemotional adversities, specifically in the area of dissociation and identity. Further studies should involve larger samples and investigate the effects of early maternal separation and disruptive caregiving on social functioning and the capacity to form and sustain close relationships in adolescence and adulthood. Ideally, we suggest employing a longitudinal design.

## Data Availability

The raw data supporting the conclusions of this article will be made available by the authors, without undue reservation.
